# Reducing friction in machine oil via cesium-hybridized graphene oxide quantum dot additives

**DOI:** 10.1038/s41598-025-30201-3

**Published:** 2025-12-10

**Authors:** Islam Gomaa, Sherif Elsoudy, Maryam G. Elmahgary, Ahmed A. Abdel-Rehim

**Affiliations:** 1 Nanotechnology Research Centre (NTRC), The British University in Egypt (BUE), Suez Desert Road, El-Sherouk City, Cairo, 11837 Egypt; 2 Department of Mechanical Engineering, Faculty of Engineering, The British University in Egypt (BUE), Suez Desert Road, El-Sherouk City, Cairo, 11837 Egypt; 3 Chemical Engineering Department, Faculty of Engineering, The British University in Egypt (BUE), Suez Desert Road, El-Sherouk City, Cairo, 11837 Egypt

**Keywords:** Cs-GOQDs, Nanolubricants, Graphene oxide quantum dots, Tribology, Energy efficiency, Friction reduction, Materials science, Nanoscience and technology

## Abstract

Friction and wear are among the most common causes of energy loss and component failure in mechanical systems, accounting for around 20–30% of total global energy consumption. Conventional lubricants fail to provide consistent performance in harsh conditions due to inadequate additive dispersion, agglomeration, and restricted surface reactivity. Recent advances in nanomaterial-based lubricants have shown promise, but it remains difficult to achieve significant friction and wear reduction at low additive concentrations while preserving stability. In this study, we synthesized and tested cesium-hybridized graphene oxide quantum dots (Cs-GOQDs) as novel lubricant additives. When incorporated into machine oil at 0.1 wt%, Cs-GOQDs reduced the coefficient of friction by 14.6% and the average wear scar diameter by 9.2%, demonstrating their effectiveness as nanolubricant additives TEM, FT-IR spectroscopy, FE-SEM, EDAX and XPS analysis revealed the production of a protective tribofilm and nanoscale rolling processes, which improved lubrication synergistically. These preliminary findings show that the innovative hybridized Cs-GOQDs provide an effective solution to overcome the limitations of conventional additives, opening up a new path for the development of next-generation nanolubricants with practical implications for energy efficiency and mechanical durability.

**Supplementary Information:**

The online version contains supplementary material available at 10.1038/s41598-025-30201-3.

## Introduction

Friction and wear account for approximately 25% of global energy consumption, resulting in more than eight gigatons of CO_2_ emissions each year^1^. As transportation and industrial activities grow, the negative effects of friction and wear on energy consumption, environmental health, the global economy, and overall sustainability are projected to intensify^2–4^. In transportation and industry, friction leads to energy inefficiency and significant economic costs from maintenance and downtime due to wear-related machinery failures; advances in tribology, featuring innovative materials and lubricants, offer promising strategies to mitigate these issues, potentially delivering substantial energy and cost savings while improving mechanical efficiency and sustainability^5–7^.

Graphene and its derivatives’ remarkable tribological properties have garnered a lot of attention. As coatings, graphene reduces friction and improves wear resistance in important engine components like pistons and bearings^8^. As a structural material, it imparts superior strength and wear resistance compared to conventional metals. The 2023 Nobel Prize in Chemistry is a significant recognition of the progress made in the synthesis and development of quantum dots (QDs) and is likely to accelerate the development of quantum dot-based technologies in recent years. Quantum scale materials (1–10 nm) are on the front of 6 G Era specially detectors^9,10^. The morphology of particle agglomerates plays an important role in improving properties such as the micro wheel array of Quantum Dots, demonstrating up to nine times more responsiveness compared with the signal of QDs films^11^.It’s worth mentioning that graphene oxide quantum dots (GOQDs) have emerged as a new class of tribological modifiers^12^. GOQDs combine high surface area, tunable electronic/optical properties, and excellent dispersion stability, making them suitable for a variety of applications in optoelectronics, catalysis, and sensing^9,13,14^. GOQDs, a preferred form of GQDs, demonstrate a significantly distinct optoelectronic nature, mostly owing to the presence of surface oxygen-rich functional groups (e.g., carboxyl or hydroxy groups).Recent studies also show that they have the potential to be used as lubricant additives, where they reduce the coefficient of friction (COF), improve efficiency, and minimize surface wear^15^, In addition cesium-based compounds exhibited self-lubricating properties open questionnaire about Cs-GQDs nanocomposite behavior^16,17^. In tribology, various nanomaterials, including graphene oxide, carbon dots, and transition metal dichalcogenides (e.g., MoS_2_), have been shown to improve lubrication through mechanisms such as nanoscale rolling, surface film formation, and tribochemical reactivity^18–20^. Notably, recent reports show increasing interest in modification of graphene for application in the field of lubrication to reduce friction and wear on moving mechanical assemblies^21–23^. These studies underline the versatility of graphene based motifs composites in lubrication science but also highlight that most research has focused on pristine or functionalized systems, with limited attention to alkali-metal-doped graphene quantum dots.

Cesium (Cs) doping is a particularly intriguing option. Cs, with its large ionic radius, low electronegativity, and strong electron affinity, can change the surface reactivity of GOQDs, aid in the production of protective tribofilms, and increase lubricating oil stability. Furthermore, Cs inclusion may improve the dispersion of GOQDs during operational conditions, increasing their tribological performance. To yet, the role of Cs doping in changing the lubricating performance of graphene-derived nanostructures has not been well studied. This research fills that knowledge gap by presenting the first synthesis and proof of concept for tribological evaluation of cesium-doped graphene oxide quantum dots (Cs-GOQDs) as lubricant additives. We show that Cs-GOQDs, even at low concentrations, considerably reduce friction and wear in lubricants, making them promising candidates for environmentally friendly formulations in internal combustion engines and electric vehicles. By integrating the particular nanoscale properties of GOQDs with the distinct physicochemical contributions of Cs doping, this study reveals a new approach for producing next-generation lubricants with both performance and environmental advantages.

## Materials and methods

### Chemicals and reagents

Citric acid (Sigma Aldrich, ≥ 99%), Cesium carbonate (Sigma Aldrich, ≥ 99%), HCl (Fiser chemical, 37%) and H_2_O_2_ (Fisher scienific, 50%), and Sodium hydroide pellets (ACS reagent, ≥ 97%), all chemicals were used without any further purifiction. The deionized (DI) Milli-Q water was used during this experiment.

### Synthesis of cesium-graphene oxide quantum dots (Cs-GOQDs)

**Fig. 1:**
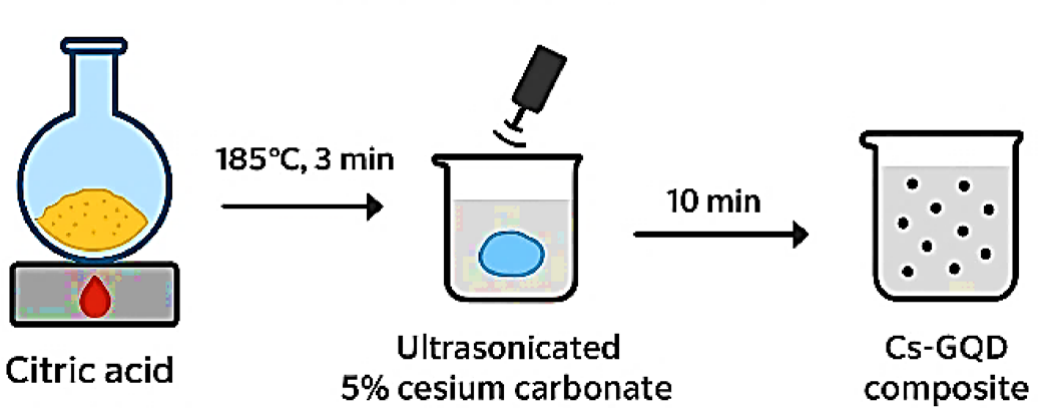
Synthesis of Cs-GOQDs steps.

Cesium-graphene-oxide quantum dots (Cs–GOQDs) and graphene-oxide quantum dots (GOQDs) were synthesized via an in-situ precursor-melting route Fig. 1. Citric acid (5.00 g) was heated to 185 °C for 3 min to generate a homogeneous molten phase. For Cs–GOQD synthesis, a 5 wt% cesium carbonate (Cs_2_CO_3_) dispersion was separately prepared by ultrasonication (20 kHz, 20 min) and dried at 120 °C for 30 min. The dried Cs_2_CO_3_ was then introduced portion wise into the molten citric acid under continuous stirring to ensure uniform incorporation. In parallel, identical processing without Cs_2_CO_3_ yielded pristine GOQDs. The resulting products were sonicated in 0.1 N NaOH for 10 min, washed with Milli-Q water, and purified by repeated centrifugation at 12,000 rpm and − 4 °C until the supernatant reached pH ≈ 6.5. Finally, both materials were dried under vacuum at 85 °C overnight, affording an orange–yellow Cs–GOQD powder and a brown GOQD powder.

### Tribological analysis

The tribological behavior of the Cs-Hybridized Graphene Oxide Quantum Dots (Cs-GOQDs) nanolubricant was evaluated using the four-ball test, following the ASTM D4172 standard. The four-ball test is a widely recognized method for assessing the friction and wear properties of lubricants. This test involves using three stationary steel balls arranged in a triangular pattern and a fourth ball, which rotates against them under a specific load, speed, and temperature. During the test, the lubricant’s performance is measured by recording the wear scar diameter on the stationary balls and the friction coefficient between the rotating and stationary balls. The results obtained from this test provide critical insights into the lubricant’s ability to reduce friction and prevent wear under simulated operational conditions. Each run is conducted based on the mentioned test parameters with three replicates. The tribological experimental procedure, conditions, and friction pairs specification in Table 1.

**Table 1:** Material properties of friction pair of balls, parameters and test conditions.

Material	Density (g/cm^3^)	Diameter (mm)	Hardness (HRC)	Poisson’s ratio	Elastic Modulus (GPa)
GCr15	7.8	12.7	63–65	0.3	208

Load (KgN),	Hertz pressure (GPa)	Rotating speeds (rpm)	Linear speed (m/s)		Temperature (°C)
40 kg ± 0.2 kg,392 *N* ± 2 N	892.1	1200	0.461		75 °C ± 2 °C

The selection of nanoparticle loading in this work follows a two-stage strategy. First, we conducted a coarse screening using near-logarithmic concentrations (0.005, 0.1, 1.0, and 1.5 wt%) to rapidly bracket a practical window.

This screening -stage spacing is appropriate where the primary goal is to verify that the synthesized nanoparticles act as effective additives initially i.e., form protective tribofilms and yield the measurable friction.

In this initial stage of the current study, our focus was placed on the controlled synthesis and preliminary tribological screening of the new nano-additives, particularly their friction-reducing capability. Comprehensive analyses of colloidal stability, dispersion behavior, and long-term lubricant performance were beyond the scope of the current study. These aspects will be addressed in the next stage of our research, where advanced characterization and optimization methods will be applied to ensure both tribological efficiency and formulation stability as in our recent studies^24,25^.

### Characterization techniques and instruments

The FTIR spectra were obtained using an FTIR spectrometer, model Vertex 70-Bruker, Germany (range 4000–400 cm^[- 1^) based on KBr discs technique. The UV-Vis absorption spectra of the samples were measured using a double beam spectrophotometer (Cary 5000 UV-Vis-NIR, Agilent Company, Malaysia). The phase composition and crystal structure examined via the X-ray diffraction (XRD), model Malvern Panalytical Empyrean (United Kingdom). XPS characterization was performed at the Center for Nanoscale Systems (CNS), Harvard University, using a Thermo Scientific K-Alpha+/Nexsa XPS System (Thermo Fisher Scientific). A monochromatic Al Kα X-ray source (hν = 1486.6 eV) was used. Survey spectra were recorded with a survey pass energy of 200 eV and high-resolution core-level spectra were recorded with a pass energy of 20 eV. The HRTEM images of the samples were determined via transmission electron microscope, operated at 200 kV, model Joel-JEM-2100 (Japan). The morphology and topology of the samples was characterized using Field-Emission Scanning Electron Microscopy (FESEM, Quattro S, Thermo Scientific, USA). High-Speed refrigerated Centrifuge High-Speed refrigerated Centrifge (Model: Hermle Z 36 HK, USA).

## Results and discussion

### Fourier transform infrared spectroscopy (FTIR)

**Fig. 2:**
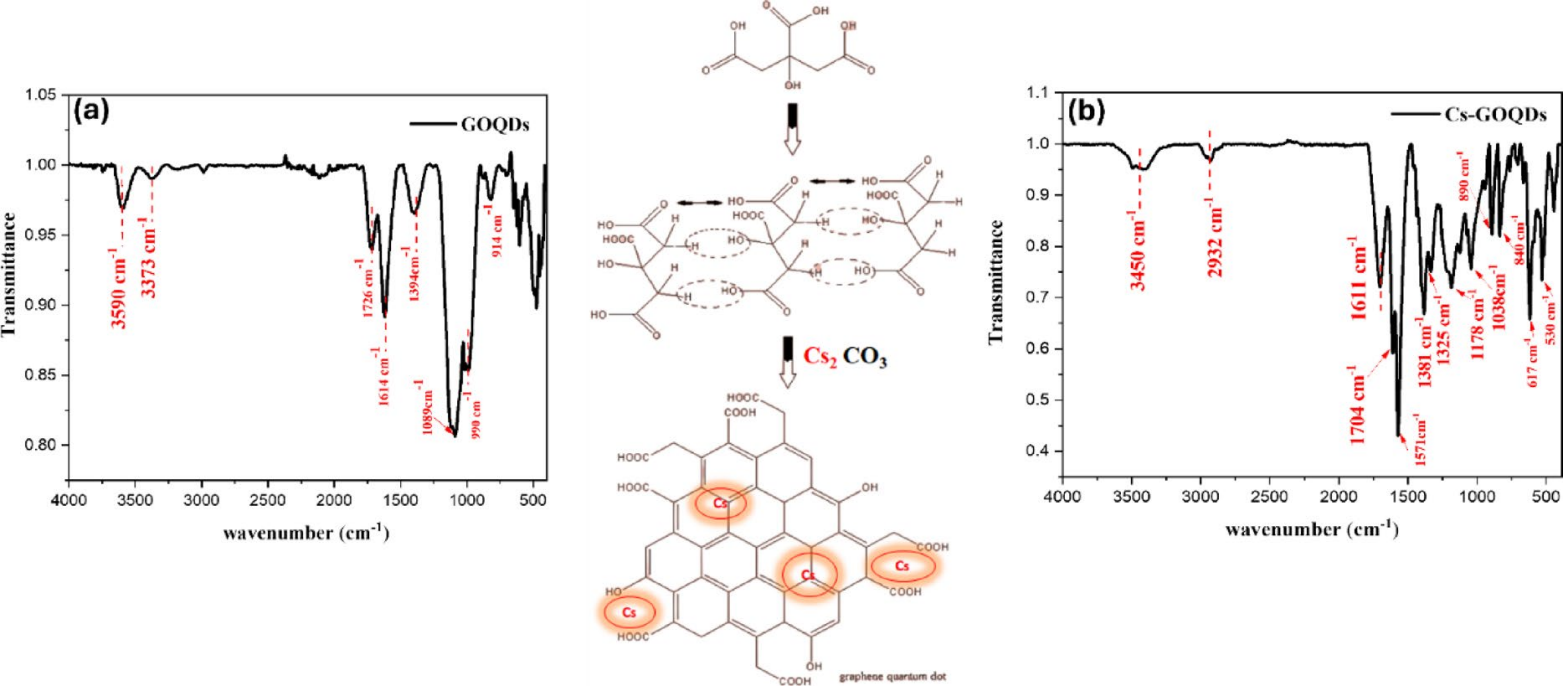
FTIR spectra and the corresponding functional group frequencies of Cs-GOQDs, along with a schematic illustration depicting the conversion process of Citric Acid to functionalized GOQDs. (**a**) GOQDs and (**b**) Cs-GOQDs aligned with schematic conversion of Citric Acid to Functionalized Cs-GOQDs.

FT–IR spectra (Fig. 2) show that thermal condensation of citric acid yields oxygen-rich, partially conjugated graphene dots whose surface chemistry is systematically modified by Cs₂CO₃ addition. Pristine GOQDs (Fig. 2a) display broad O–H bands at ≈ 3590 and ≈ 3373 cm⁻¹ and a strong C = O resonance at 1726 cm⁻¹, consistent with abundant –COOH/ester moieties. The feature at ≈ 1614 cm⁻¹ reports the emergence of sp² C = C character and/or asymmetric COO⁻ vibrations, while the band at 1394 cm⁻¹ corresponds to symmetric COO⁻/O–H bending^9,26^. Intense C–O vibrations occur near 1089 cm⁻¹ with a shoulder at ≈ 990 cm⁻¹ and a minor band at 914 cm⁻¹, indicative of alcohol/ether/epoxide and minor cyclic-oxygen fragments formed during melt condensation^27–31^. Addition of Cs (Fig. 2b) preserves these vibrational families but produces reproducible spectral shifts and intensity changes that are diagnostic of direct Cs–oxygen interactions and partial deprotonation of surface acids. The carbonyl band downshifts (1726 → ≈1704 cm⁻¹) and the O–H envelope broadens and shifts to lower wavenumber (center ≈ 3450 cm⁻¹), consistent with coordination of Cs⁺ to carbonyl and hydroxyl oxygens (coordination weakens the C = O bond and alters H-bonding/ion-pairing). In the fingerprint region C–O/COO features redistribute (notably peaks near 1325 and 1038 cm⁻¹ intensify relative to the GOQD 1394/1089 pattern), indicating a changed balance of ether/ester/epoxide species and an increased fraction of deprotonated carboxylates. New or enhanced low-frequency bands at ≈ 890 and 840 cm⁻¹, together with subtle features near 617 and 530 cm⁻¹, likely reflect lattice-coupling or metal–oxygen/metal–carbon modes associated with Cs incorporation^32^.

### X-ray diffraction and X-ray photoelectron spectroscopy analysis

XRD patterns of GOQDs and Cs–GOQDs (Figure S4a) reveal both shared graphitic signatures and distinct features introduced by cesium incorporation. The GOQDs display reflections at 2θ ≈ 7.7°, 26.6° and 43.8°, assignable to the (111), (002) and (100) planes, respectively, which correspond to d-spacings of ≈ 14.30 Å, 3.38 Å and 2.03 Å^9^. The (002) reflection is shifted to lower angle relative to bulk graphite (d ≈ 3.34 Å), indicating enlarged interlayer separation. This expansion is readily explained by the high degree of surface oxidation (–OH, –COOH, epoxide) and by intercalated water molecules that increase the average layer-to-layer distance and disrupt ideal π–π stacking, consistent with a partially ordered, oxygen-rich graphitic network^9^. In the Cs–GOQDs composite the diffraction pattern retains a low-angle graphitic feature (≈ 7.5°) but additionally exhibits a broad hump across 20–40° and multiple sharper reflections at 2θ = 7.58°, 15.37°, 17.10°, 18.84°, 24.23°, 25.99°, 30.93° and 38.71°, with corresponding d-spacings of ≈ 11.65, 5.76, 5.18, 4.71, 3.67, 3.43, 2.89 and 2.32 Å. The low-angle and ~ 28.7° features are consistent with graphitic carbon, while the series of additional peaks are attributable to cesium-containing species: several reflections match reported signatures of Cs-carbon clusters (e.g., Cs_6_C_60_) as referenced by 01–079-1713 (Figure S1), and the sharp high-angle signal near 38.7° is indicative of highly localized cesium sites or very small Cs crystallites^33,34^. The appearance and slight displacement of these peaks relative to reference positions suggest that cesium incorporation produces local lattice perturbations and residual stress in the carbon framework. Furthermore, the peak at ~ 30.9° is consistent with cesium–oxygen cluster phases (e.g., cesium ozonide) that can form when alkali atoms interact with oxygen-rich GOQD surfaces. Taken together, the data indicate that cesium is present both as dispersed atomic/cluster species interacting with the GOQD matrix and as small, well-defined crystalline domains, and that its presence increases structural heterogeneity and interlayer spacing compared with pristine GOQDs^35,36^. Quantitative crystallite metrics (average crystallite size D, dislocation density δ, and microstrain ε) were obtained using the Scherrer relation (Eq. 1) and the standard relations for δ and ε (Eqs. 2–3), allowing direct of the degree of ordering and defect density of Cs–GOQDs nanocomposite^37^.

1 $$\:D\:\left(crystallite\:size\right)\:in\:nm=\left[\frac{\left(k\right)\times\:\left(\lambda\:\right)}{\left({\beta\:}_{D}\right)\times\:\left(Cos\theta\:\right)}\right]\:\:\:$$

2 $$\delta ~ = ~{\raise0.7ex\hbox{$1$} \!\mathord{\left/ {\vphantom {1 {D^{2} }}}\right.\kern-\nulldelimiterspace} \!\lower0.7ex\hbox{${D^{2} }$}}$$

3 $$~~\varepsilon ~ = ~\beta ~\cos \theta \backslash 4$$

The crystallite size was estimated using the full width at half maximum (FWHM) of the 2θ peak at 7.5 degrees, resulting in a calculated particle size of 5.5 nm (details in Table 2). Further analysis revealed dislocation densities and micro-strain of the Cs-GOQDs to be 0.0326 and 0.0655, respectively. These values indicate a crystalline structure with a minimal amount of defects and distortions within the Cs-GOQDs nanocomposite^38^; moreover give good indication of stability and decline of crystal entropy and free energy^39,40^.

The XPS analysis, as depicted in Figure S4b-c, provides valuable insights into the electronic structure and chemical composition of the nanocomposite. The peak observed at 284 eV in Figure S4b corresponds to the delocalization of sp² hybridized carbon atoms (aromatic carbon)^41,42^. This observation signifies the presence of a graphene-like resonant structure within the nanocomposite. The broader peak at 289 eV can be attributed to the presence of carboxylic moieties (COOH groups) bound to the carbon edge framework^43^. Figure S4c reveals the presence of cesium atoms in the nanocomposite, the peaks at 724 eV and 730 eV correspond to the spin-orbit split 3 d electrons of cesium, specifically the 3 d (5/2) and 3 d (3/2) subshells, respectively. Notably, a prominent peak is observed at 532.3 eV in Figure S4c, which can be assigned to the O 1 s core level electrons, oxygen in hydroxyl groups (O-H) bonded to the surface can contribute to a peak around 531–532 eV, potentially overlapping with the peak from cesium-oxygen bonds. So binding energy suggests the formation of stable chemical bonds between oxygen and cesium atoms within the nanocomposite (Oxygen-Cesium binding)^44,45^.

**Table 2:** The crystal structure parameters of Cs-GOQDs.

Sample name	(hkl)	d-spacing	β_D_ (rad)	D (Scherrer eq.) (nm)	δ (nm ^2^)	ε
Cs-GOQDs	(111)	10.96	0.02515	5.5	0.032610252	0.065503

### FESEM, EDAX, and HRTEM analysis

**Fig. 3:**
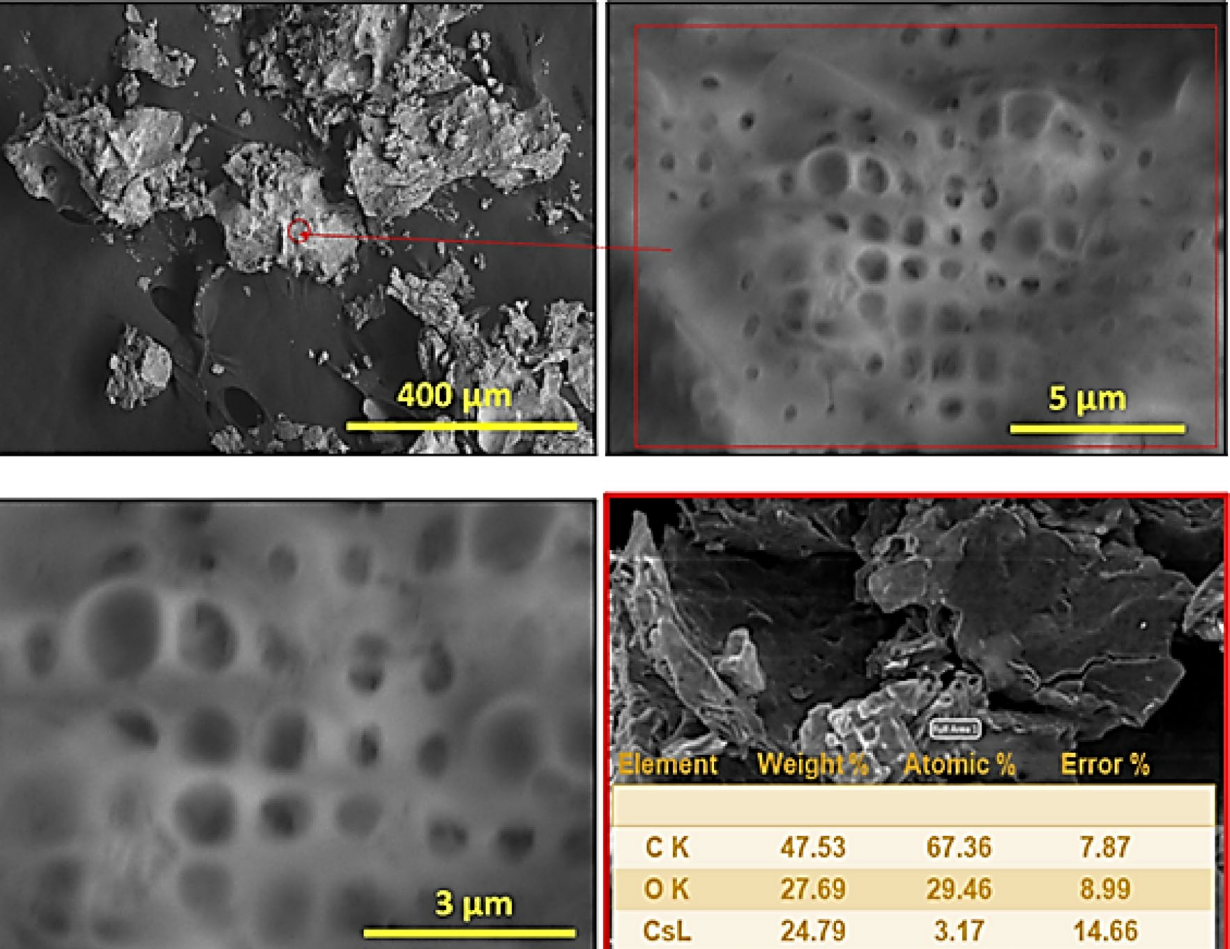
The Morphological structure of Cs-GOQDs. High-magnification FESEM reveals sponge-like porous structures (20–500 nm) aligned with EDAX analysis.

Cs-GOQDs nanocomposites examined through FESEM in Fig. 3. These Cs-GOQDs appears to be confluence of intermolecular forces such as Van der Waals interactions The hydrophobicity of GOQDs and the low aqueous affinity of Cs^+^ cations promote the aggregation of hydrophobic domains^46^. Favorable π-π stacking interactions between the aromatic carbon rings of GOQDs and chemisorbed Cs^+^ further engender layered structures, along with electrostatic bridging by Cs^+^ cations between negatively charged oxygen moieties, promote layered aggregation. Hydrogen bonding between oxygen-containing groups further enhances cluster formation. High-magnification FESEM reveals sponge-like porous structures (20–500 nm), likely due to gas evolution during synthesis^47^. The Energy-dispersive X-ray spectroscopy (EDAX) surface analysis revealed elemental weight percentages of 47.5% carbon, 27.6% oxygen, and 24.7% cesium, with corresponding atomic percentages of 67.3% carbon, 29.4% oxygen, and 3.17% cesium. These values align closely with the calculated mass percentages (C 47.5% and Cs 52.5%) and atomic percentages (C 90.9% and Cs 9.1%) for Cs_6_C_60_, suggesting minimal impurities and a successful approximation of the molecular formula. The notable disparity between the experimental cesium percentage and the calculated value for Cs_6_C_60_ can be attributed to the presence of oxygen moieties within the formula. Nevertheless, the close agreement between measured and theoretical values underscores a well-distributed elemental presence across the sample surface and stoichiometry at the molecular level. Figure S2 presents high-resolution elemental mapping of Cs-GOQDs obtained using EDAX. The full-surface mapping (panel a) reveals a well-distributed network of carbon (C) and oxygen (O) represented by densely packed points, indicative of a rich crystalline phase for both elements. Notably, the cesium (Cs) mapping (panel c) demonstrates a distinct feature – isolated bright spots scattered across the surface. These individual spots, unlike the clustered distribution observed for C and O, suggest the presence of discrete Cs clusters rather than larger agglomerates. The discernible isolated bright spots evident in EDAX mapping of Cs-GOQDs as illustrated in Figure S2, potentially signify the integration of singular cesium atoms within the graphene oxide matrix. This proposed explanation stands as a more plausible conjecture when juxtaposed with the notion of single crystals for several compelling reasons. Firstly, the characteristic size of singular cesium atoms markedly contrasts with the observed features in the mapping, aligning seamlessly with the distinct isolated bright spots discerned therein. Such congruence underscores the feasibility of singular cesium atom integration within the graphene oxide lattice. Secondly, the viability of this proposition is further corroborated by considerations of synthesis methodology. Depending on the intricacies of the specific synthetic route employed for Cs-GOQDs fabrication, it remains conceivable to attain a degree of dispersion wherein cesium atoms are individually tethered onto the GOQDs substrate. Such controlled synthesis processes offer a conducive milieu for the precise incorporation of singular cesium atoms, thereby lending credence to the plausibility of their presence within the Cs-GOQDs structure. Elemental Analysis aligns perfectly with the well-distributed elemental composition identified through bulk EDAX analysis, confirming the uniform presence of C, O, and Cs throughout the Cs-GOQD sample. Figure S3 showcases the utility of scanning transmission electron microscopy (STEM) in elucidating the agglomeration behavior of Cs-GOQDs within water. The image reveals a pronounced tendency for the quantum composite dots to aggregate into spherical clusters, appearing as distinct dark spots. This observation underscores the importance of considering potential agglomeration phenomena when studying the interactions between Cs-GOQDs and various solvent environments. Cs-GOQDs exhibit a propensity to aggregate into spherical clusters, driven by mechanisms that optimize their energy state. This aggregation phenomenon is orchestrated by a multifaceted interplay of factors. Firstly, the coalescence into spherical clusters minimizes the total surface area exposed to the solvent, thereby reducing interfacial energy and achieving a more energetically favorable configuration. Weak Van der Waals interactions between aromatic carbon rings intensify as Cs-GOQDs approach each other, facilitating the formation of compact, spherical clusters. Additionally, π-π stacking interactions between GOQDs and chemisorbed Cesium foster a layered structure conducive to spherical arrangement. Despite potential favorable interactions with solvent functional groups, the nature of GOQDs leads to unfavorable interactions with solvent molecules, driving aggregation to minimize solvent exposure. Cs-GOQDs, undergoing continuous Brownian motion in a liquid environment, collide with solvent molecules, enabling them to adhere to one another and form stable spherical clusters. In summary, the aggregation of Cs-GOQDs into spherical clusters represents a complex interplay of surface area minimization, intermolecular interactions, and dynamic motion within the solvent environment, ultimately resulting in the formation of energetically favorable configurations.

**Fig. 4:**
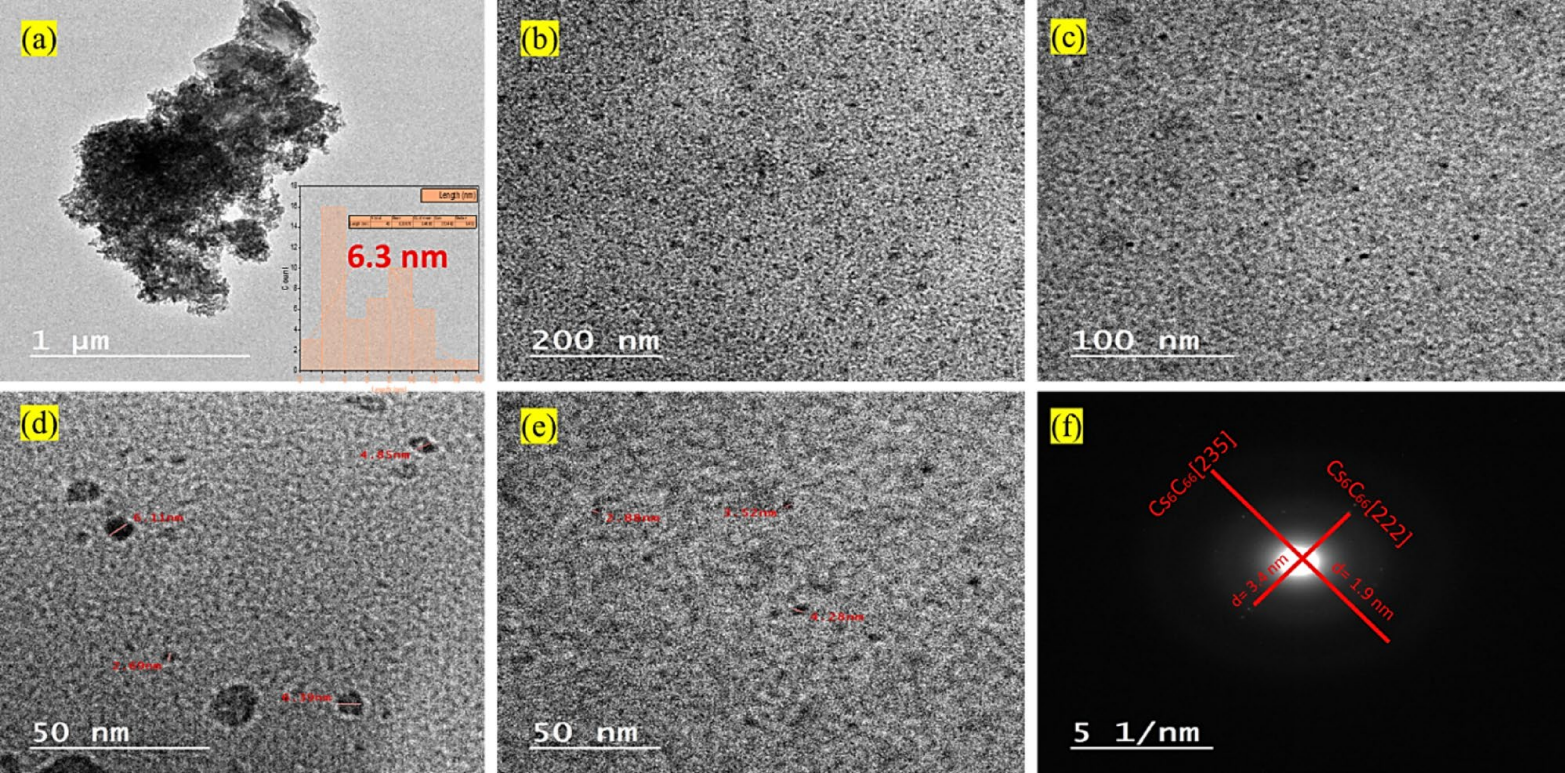
HRTEM images of individual Cs-GOQD particles (panels a-e). Complemented by a selected area electron diffraction (SAED) pattern (panel f).

Aqueous pre-processing employing vigorous sonication facilitates the deconstruction of agglomerated Cs-GOQDs clusters, enabling subsequent analysis using HRTEM at an accelerating voltage of 200 kV, Fig. 4a vividly portrays the pronounced propensity of these minuscule, dark particles to aggregate, forming densely packed clusters. These clusters, characterized by high agglutination, generate voids and pores of varying dimensions throughout the entire structure. Delving deeper into the particle size and morphology of Cs-GOQDs (Fig. 4b-e), they manifest as obscure regions exhibiting quasi-spherical and elliptical geometries. These nanoparticles are randomly dispersed and appear as distinct spots on the grid, exhibiting a size distribution ranging from 1 to 11 nm. The average particle size was determined by statistically evaluating a multitude of particles from the TEM images using ImageJ software. This analysis yielded a dominant particle size of approximately 6.3 nm, indicative of a substantial presence of defects and imperfections within the crystalline framework. Furthermore, HRTEM images of isolated Cs-GOQDs (Fig. 4d) unveil the existence of disordered edges and non-systematic surfaces. Selected area electron diffraction (SAED) analysis (Fig. 4f**)** elucidates the polycrystalline nature of the composite, characterized by the presence of two prominent planes with d-spacings of 1.9 nm and 3.4 nm, corresponding to the (235) and (222) reflections of the cesium carbide (Cs_6_C_60_) cubic phase, as corroborated by reference code 01–079-1713 (PDF S1)^33^.

### Tribological behavior of Cs-GOQDs

Nanolubricants have garnered significant attention in recent years due to their potential to significantly reduce the COF in various applications as in electrical and hybrid vehicles and wind turbine energy^48^. This is particularly critical in enhancing the efficiency and longevity of mechanical systems. Recent studies have explored a variety of nanoparticles as additives in lubricants, demonstrating notable reductions in friction and wear as shown in Table 3.

**Table 3:** Recent literature of nano additives contribution to tribological behavior of base lubricants.

Year	Nano additives	Testing conditions/concentration	Percentage reduction in COF	Reference	
2023	Al_2_O_3_-SiO_2_/DEC PAG	Four-ball, ASTM D4172: 40 kg load, 1200 rpm, 75 °C, 60 min.	8.1%	^49^	
2022	ZrO_2_/GO hybrid	Block-on-ring (stainless/alloy steel), 20 N, 300 mm·s⁻¹, ~ 25 °C.	57%	^50^	
2023	TiO_2_-SiO_2_/POE	Four-ball, ASTM D4172: 1200 rpm, 40 kg, 75 °C, 60 min.	31.6%	^51^	
2022	Graphene nanolubricant	Pin-on-disk, WC–Co pin/TC4 or 6061 disk; 2 N, 50 mm·s⁻¹; water or 10% isopropanol carrier.	29%	^52^	
2022	Carbon dots	Ball-on-disk; tested in water & sunflower oil; loads 2–3 N (examples below).	75%	^53^	
2022	Graphene-enhanced nanolubricant	Four-ball per ASTM (tribology + viscosity per ASTM methods).	10.4%	^54^	
2023	Nitrogen-doped carbon dots	(Ball-on-disk) friction stable ~ 5 h; base oils: sunflower (SFO) & PAO10.	40%	^55^	
2025	Cs-GOQD	Four-ball, 0.1 wt%, 392 N, 75 °C	14.6%	Current work	

**Fig. 5:**
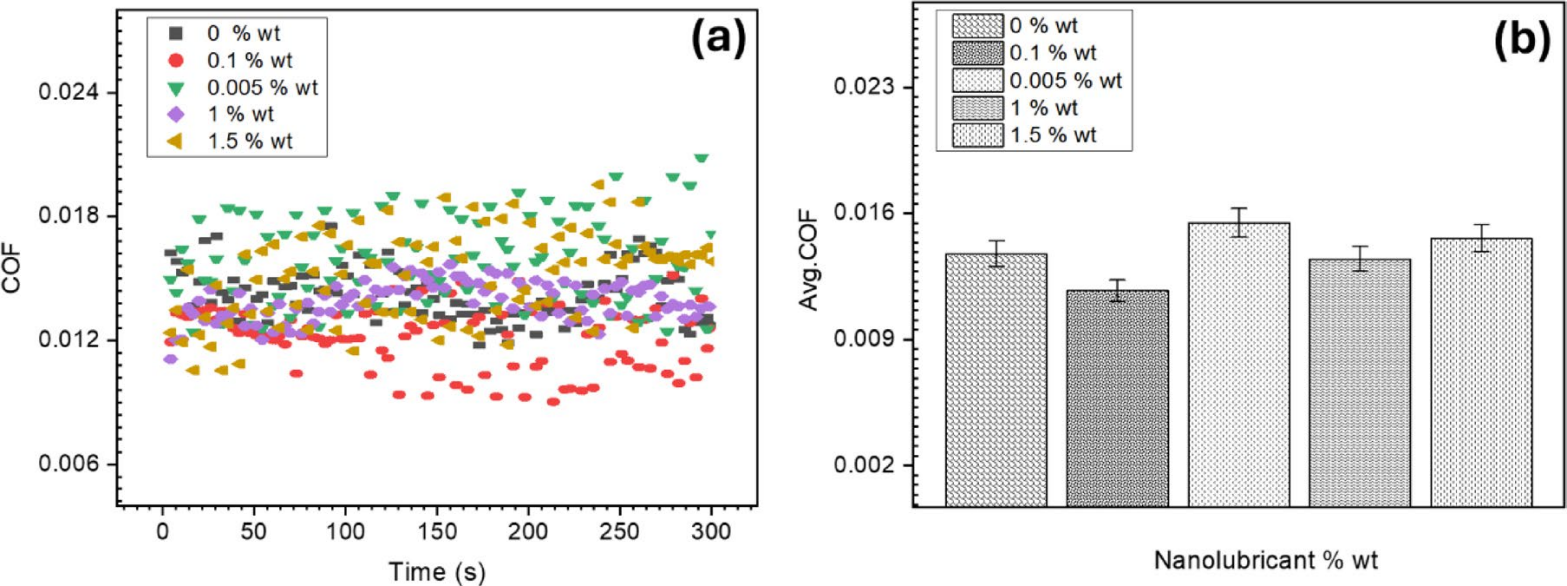
Four ball test results, Cs-GOQDs nanocomposite at a concentration of 0.1 wt% demonstrated remarkable consistency in reducing COF, (**a**) COF with sliding time, (**b**) Mean COF.

**Fig. 6:**
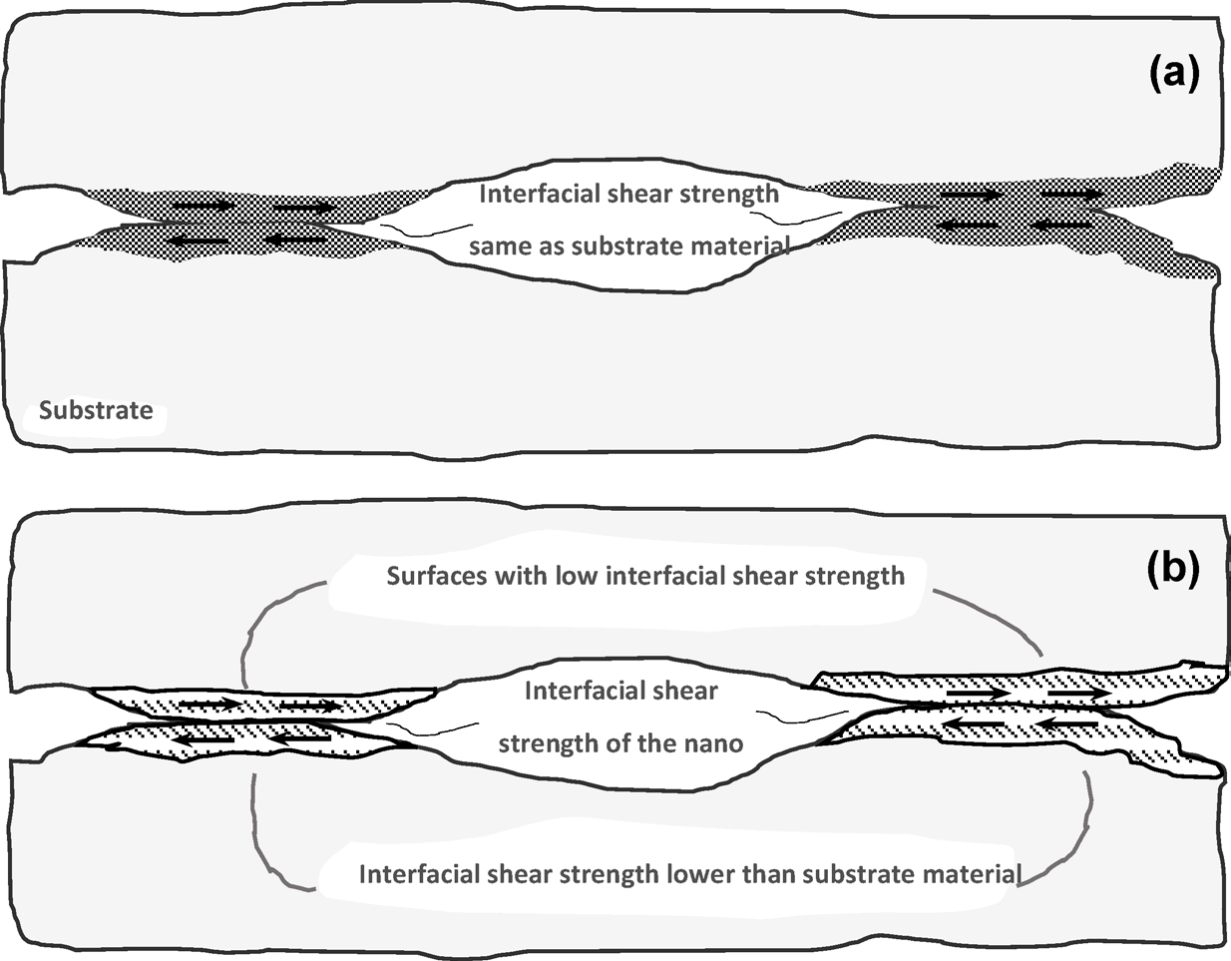
Boundary Lubrication at atomic level of contact asperities (**a**) High-shear-strength by surface asperity summits of substrate material, (**b**) Low-shear-strength layer formed by nano tribo-films at asperity summits.

In the current results and analysis section, we conducted a four-ball test to initially verify the anti-friction and anti-wear performance of nanolubricants, a crucial aspect highlighted in recent research^48,56^. Figure 5a-b illustrates that the inclusion of 0.1 wt% concentration resulted in a noteworthy 14.6% reduction in the COF compared to the control samples. The dynamic COF fluctuations observed in Fig. 5a are attributed to the energy required for shearing surface asperities during relative motion between sliding surfaces. Specifically, Cs-GOQDs nanocomposite at a concentration of 0.1 wt% demonstrated remarkable consistency in reducing COF (Fig. 5a). The remarkable 14.6% reduction in COF observed with Cs-GOQDs can likely be attributed to a synergistic combination of factors. The presence of Cs-GOQDs within the lubricant is believed to promote the formation of a protective tribofilm on the interacting surfaces during boundary lubrication. This film acts as a barrier, separating asperities (microscopic high points) and minimizing direct contact with reducing the interfacial shear strength of surface asperities as hypothesized in many recent studies regarding nano lubrication mechanisms^24^ Figure 6. Additionally, the spherical or quasi-spherical morphology of individual Cs-GOQDs, as revealed by HRTEM analysis, could contribute to a rolling effect at the contact interface. These Cs-GOQDs might function like miniature ball bearings, facilitating a rolling motion between asperities and reducing friction compared to direct shearing. Furthermore, functional groups (C-O, C = C, and OH) confirmed by FTIR spectroscopy might enhance lubricity by interacting with the lubricant or surfaces, creating a more lubricious interface. Finally, cesium atoms within the Cs-GOQDs hold the potential to participate in tribochemical reactions at the contact interface. Whilst the exact nature of these reactions, they could involve the formation of protective surface layers or the modification of contacting surfaces to reduce friction.

However, deviating from this optimal concentration proved counterproductive. Lowering the concentration to 0.005 wt% or hypothetically increasing it up to 1.5 wt% led to a degradation in the lubrication mechanism of the nanolubricant. This hypothetical degradation is tentatively linked to nanoparticles potentially acting as third-body abrasion wear at certain concentrations, aligning with findings in relevant studies^57^.

**Fig. 7:**
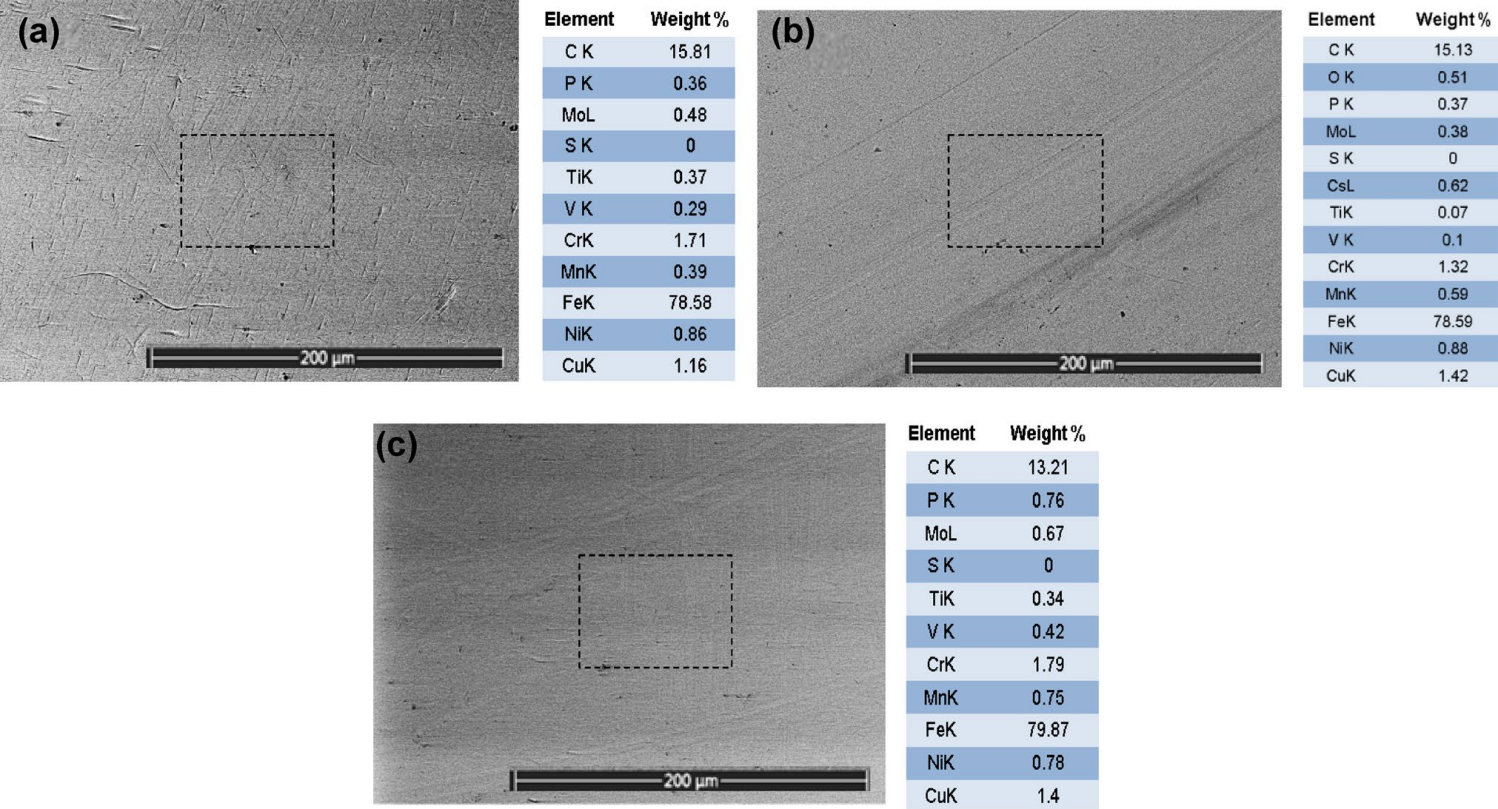
Morphological SEM micrographs with EDAX mapping analysis of Balls integrated with FBT test, (**a**) Blank sample of new ball, (**b**) Nanolubricant 0.1% wt. (**c**) Bare lubricant- 0%wt.

The primary justification for this improvement in tribological performance of the lubricant could be attributed to many reasons. The primary lubrication mechanism involves forming a physical barrier that minimizes direct contact between surfaces, thereby reducing friction and wear. Figure 7b shows the formation of Cs tribofilm at surface asperities compared to the Bare sample Figure 7c. For Cesium, Cesium’s high atomic weight and large ionic radius contribute to creating a stable lubricating film, reducing metal-to-metal contact as shown in SEM/EDAX analysis Figure 7. Cs nanoparticles exhibit high chemical reactivity, which facilitates the formation of a tribofilm on the surface of the friction pairs. This tribofilm acts as a protective layer, reducing wear. Due to its higher atomic mass, Cs nanoparticles can effectively distribute the applied load over a larger contact area, minimizing localized stress points and reducing wear.

On the other hand, graphene oxide quantum dots are gaining popularity due to their exceptional mechanical strength, thermal conductivity, and chemical stability. These properties make them excellent candidates for reducing friction and wear in lubricants. The primary mechanisms include the rolling mechanism, where graphene dots act like nano ball bearings, reducing friction by rolling between contact surfaces, and the formation of a protective tribo-film on the surface, which reduces direct metal contact and wear^58^.

On the other hand, graphene oxide quantum dots are gaining popularity due to their exceptional mechanical strength, thermal conductivity, and chemical stability. These properties make them excellent candidates for reducing friction and wear in lubricants. The primary mechanisms include the rolling mechanism, where graphene dots act like nano ball bearings, reducing friction by rolling between contact surfaces, and the formation of a protective tribo-film on the surface, which reduces direct metal contact and wear^58^.

The presence of oxygen-containing functional groups on GOQDs enhances their dispersion stability in lubricants and improves their ability to form strong bonds with the base oil and the metal surfaces. These functional groups also contribute to the formation of a continuous and stable lubricating layer. Furthermore, graphene’s high thermal conductivity helps in dissipating heat generated during friction, thereby protecting the lubricated surfaces from thermal degradation. Studies have demonstrated that carbon quantum dots show excellent friction-reducing and anti-wear properties due to their small size and oxygen-containing functional groups^59^. Comparing to literature, by leveraging these properties, Cs-GOQDs hybrid nanolubricants offer significant improvements in reducing friction and wear, enhancing the overall performance and longevity of mechanical systems. The observed enhancement of 14.6% in the current study can be primarily attributed to the unique combination of these physical and chemical characteristics, which work together to provide superior lubrication and protection.

## Conclusion

In conclusion, this study demonstrates that Cs-GOQDs nanocomposite lubricants significantly reduce friction in machine oil, with indirect evidence such as reduced wear scar diameter and surface analysis suggesting potential improvements in wear resistance. The four-ball test showed that incorporating 0.1 wt% Cs-GOQDs led to a 14.6% reduction in the COF compared to control samples. This improvement is primarily attributed to the formation of a protective tribofilm that minimizes direct asperity contact and the rolling effect of quasi-spherical nanoparticles, which act like nano ball bearings. Additionally, functional groups (C–O, C = C, and O–H) enhance lubricity by interacting with the base lubricant and metal surfaces. The high atomic weight of cesium contributes to a stable lubricating film, effectively distributing the applied load and reducing localized stress. However, deviations from the optimal concentration adversely affected lubrication performance, likely due to nanoparticle agglomeration and abrasive effects. These findings highlight the importance of precise concentration optimization for achieving superior anti-friction and anti-wear properties. Overall, the integration of Cs-GOQDs into lubricants offers a promising approach for reducing friction, enhancing energy efficiency, and extending the lifespan of mechanical systems, paving the way for next-generation high-performance industrial lubricants. While the present study reports standard ASTM D4172 four-ball tribological results, full characterization of load-carrying capacity (e.g., variable-load contact studies, extreme-pressure, last non-seizure and sintering-load tests) are recommended for future work to validate and extend the present findings alongside with full dispersion stability.

## Supplementary Information

Below is the link to the electronic supplementary material.

Supplementary Material 1

## Data Availability

Data is provided within the manuscript and supplementary information files.
