# Supplementary material for: Reducing friction in machine oil via cesium-hybridized graphene oxide quantum dot additives
Source: Sci Rep. 2025 Dec 10;15:43584. doi: 10.1038/s41598-025-30201-3 (PMC12698670; doi:10.1038/s41598-025-30201-3)
Supplement: Supplementary file 1 — Supplementary Material 1 [file 41598_2025_30201_MOESM1_ESM.docx]

**Reducing Friction in Machine Oil via Cesium-Hybridized Graphene Oxide Quantum Dot Additives**

Islam Gomaa^1^, Sherif Elsoudy^2^, Maryam G. Elmahgary^3^*, Ahmed A. Abdel-Rehim^2^

**^1^** *Nanotechnology Research Centre (NTRC), The British University in Egypt (BUE), Suez Desert Road, El-Sherouk City, Cairo, 11837, Egypt.*

**^2^** *Department of Mechanical Engineering, Faculty of Engineering, The British University in Egypt (BUE), Suez Desert Road, El-Sherouk City, Cairo, 11837, Egypt.*
 **^3^** *Chemical Engineering Department, Faculty of Engineering, The British University in Egypt (BUE), Suez Desert Road, El-Sherouk City, Cairo, 11837, Egypt.*

*Corresponding author: Maryam G. Elmahgary (e-mail: [maryam.galal@bue.edu.eg](mailto:maryam.galal@bue.edu.eg)).

***Supplementary Information***


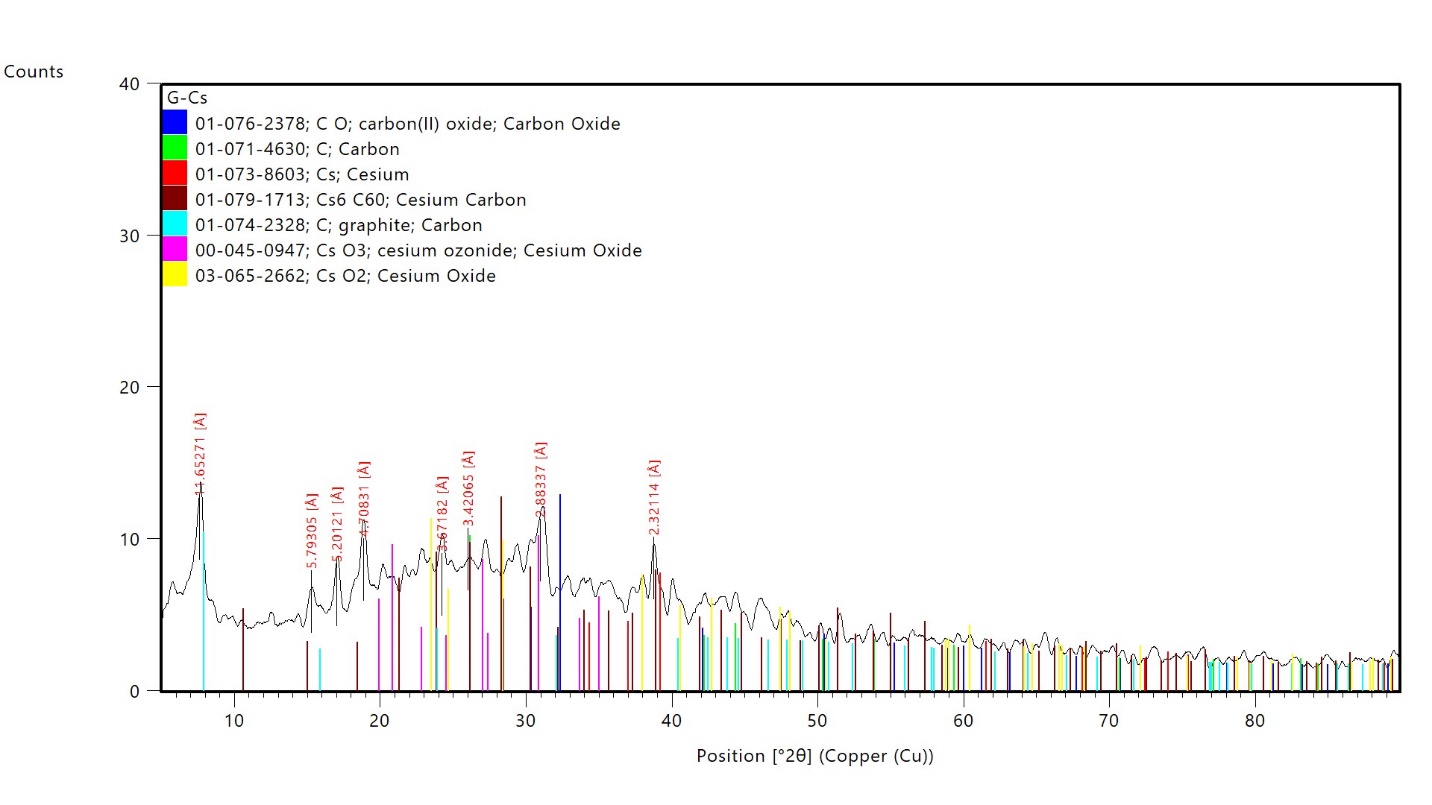

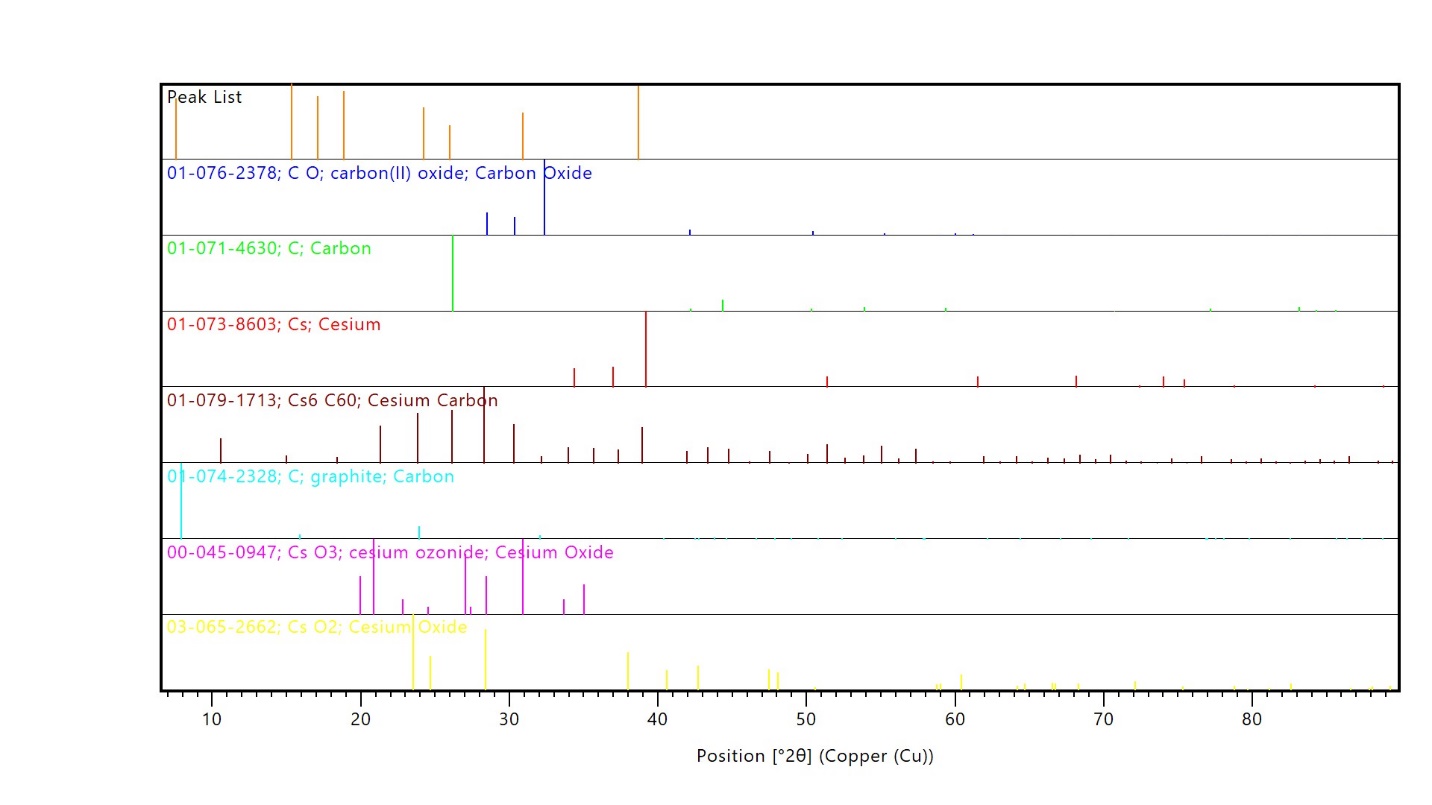


**Figure S1.** Comparison of Cs-GOQDs XRD Peaks to ICDD Reference Cards.


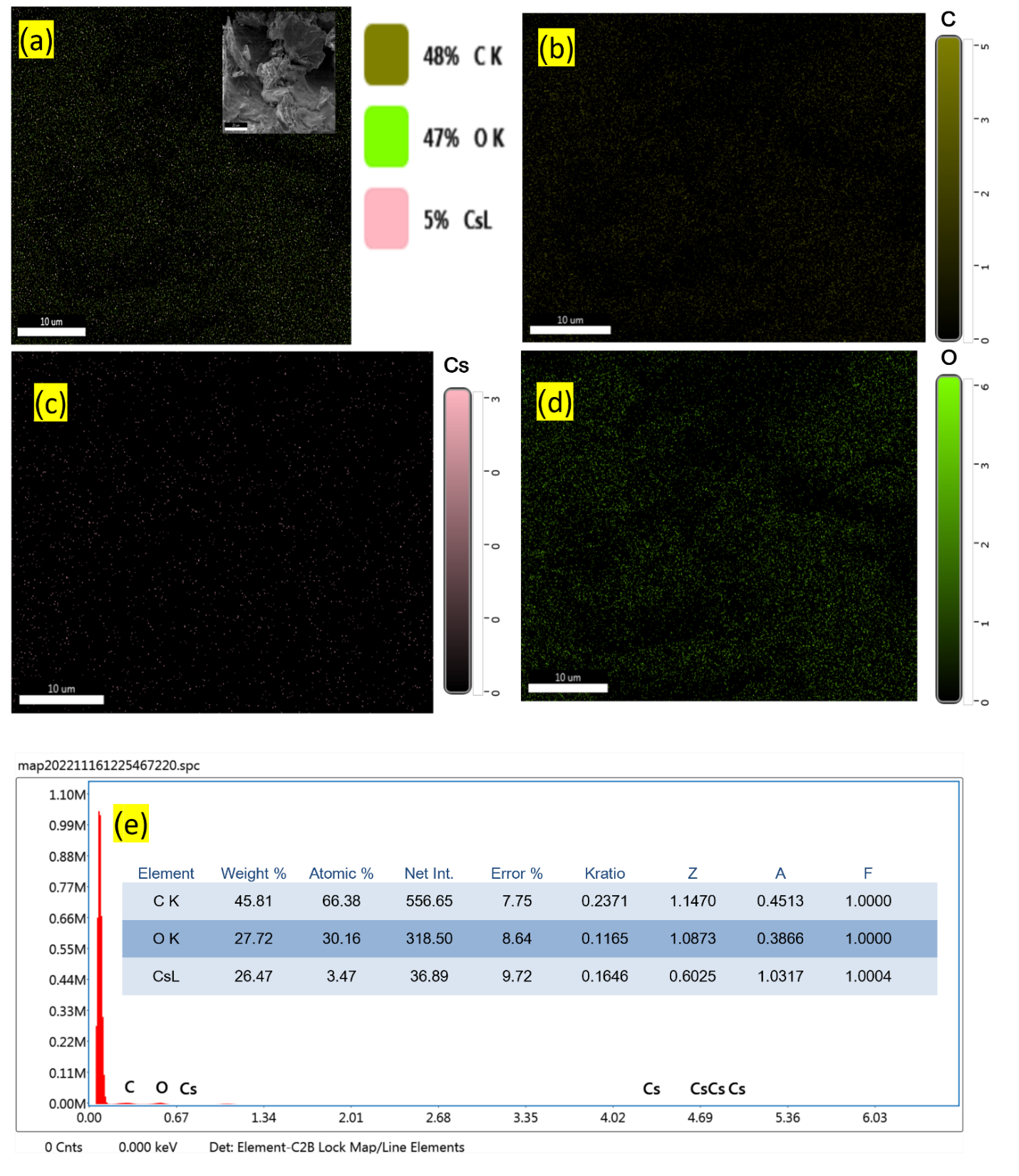


**Figure S2.** Energy-Dispersive X-ray Spectroscopy (EDAX) Analysis and Elemental Mapping of Cs-GOQDs. (a) Full-surface elemental mapping of carbon (C), cesium (Cs), and oxygen (O), (b) Individual elemental map for carbon distribution, (c) Individual elemental map for cesium distribution and (d) Individual elemental map for oxygen distribution.


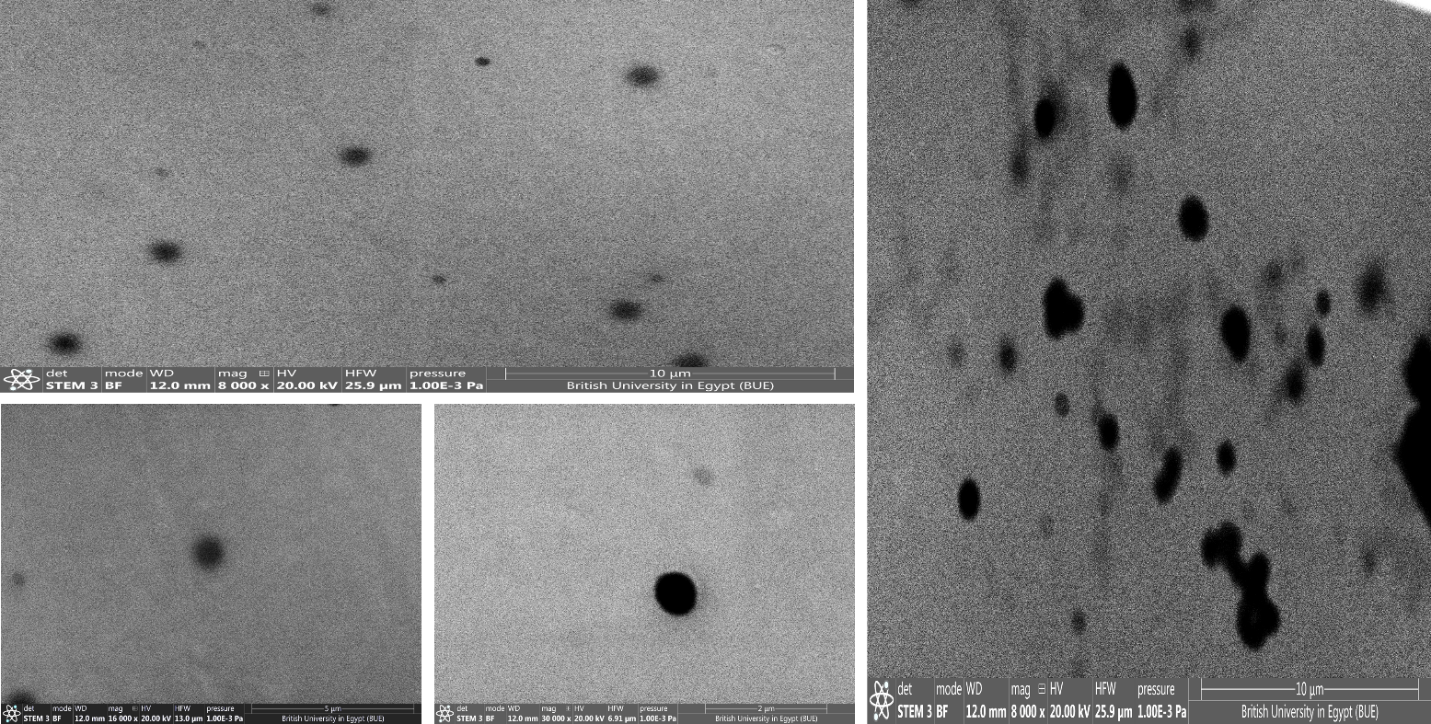


**Figure S3.** STEM of Cs-GOQDs Surface images at different magnification


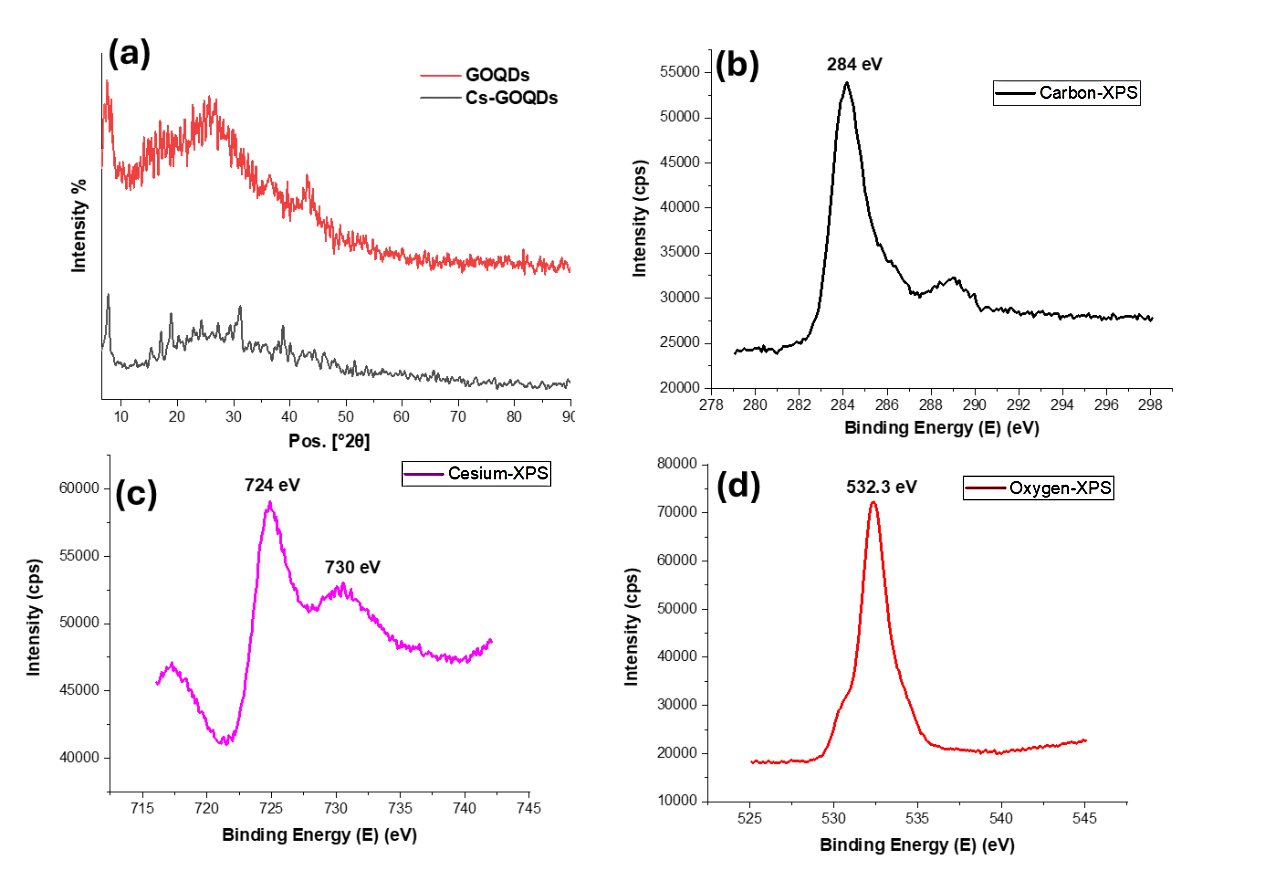


**Figure S4.** Structural and surface-chemical characterization of GOQDs and the Cs–GOQDs nanocomposite. (a) X-ray diffraction (XRD) patterns of pristine GOQDs and the Cs–GOQDs nanocomposite, together with the X-ray photoelectron spectroscopy (XPS) survey spectrum for the Cs–GOQDs nanocomposite; (b) XPS C 1s spectrum; (c) XPS Cs 3d spectrum; (d) XPS O 1s spectrum.
